# Bilayer 3D co‐culture platform inducing the differentiation of normal fibroblasts into cancer‐associated fibroblast like cells: New in vitro source to obtain cancer‐associated fibroblasts

**DOI:** 10.1002/btm2.10708

**Published:** 2024-08-05

**Authors:** Yeon Ju Kim, Hyeon Song Lee, Dohyun Kim, Hwa Kyung Byun, Woong Sub Koom, Won‐Gun Koh

**Affiliations:** ^1^ Department of Radiation Oncology, Yonsei Cancer Center Yonsei University College of Medicine Seoul South Korea; ^2^ Department of Chemical and Biomolecular Engineering Yonsei University Seoul South Korea; ^3^ Department of Radiation Oncology, Yongin Severance Hospital Yonsei University College of Medicine Yongin South Korea

**Keywords:** 3D co‐culture, bilayer hydrogel, cancer‐associated fibroblasts, fibroblast activation, radio‐ and chemoresistance

## Abstract

This study presents a novel in vitro bilayer 3D co‐culture platform designed to obtain cancer‐associated fibroblasts (CAFs)‐like cells. The platform consists of a bilayer hydrogel structure with a collagen/polyethylene glycol (PEG) hydrogel for fibroblasts as the upper layer and an alginate hydrogel for tumor cells as the lower layer. The platform enabled paracrine interactions between fibroblasts and cancer cells, which allowed for selective retrieval of activated fibroblasts through collagenase treatment for further study. Fibroblasts remained viable throughout the culture periods and showed enhanced proliferation when co‐cultured with cancer cells. Morphological changes in the co‐cultured fibroblasts resembling CAFs were observed, especially in the 3D microenvironment. The mRNA expression levels of CAF‐related markers were significantly upregulated in 3D, but not in 2D co‐culture. Proteomic analysis identified upregulated proteins associated with CAFs, further confirming the transformation of normal fibroblasts into CAF within the proposed 3D co‐culture platform. Moreover, co‐culture with CAF induced radio‐ and chemoresistance in pancreatic cancer cells (PANC‐1). Survival rate of cancer cells post‐irradiation and gemcitabine resistance increased significantly in the co‐culture setting, highlighting the role of CAFs in promoting cancer cell survival and therapeutic resistance. These findings would contribute to understanding molecular and phenotypic changes associated with CAF activation and provide insights into potential therapeutic strategies targeting the tumor microenvironment.

**Translational Impact Statements**

This study introduces a novel 3D in vitro bilayer hydrogel‐based co‐culture platform that successfully mimics the tumor microenvironment and facilitates the transformation of normal fibroblasts into cancer‐associated fibroblasts (CAFs). Our platform offers a stable and continuous source of CAFs, providing a superior CAF‐transformation effect compared to the 2D co‐culture platform. Our findings underscore the crucial role of CAFs in enhancing tumor resistance to chemo‐ and radiotherapies, positioning our platform as a potentially useful platform for developing targeted therapies against this CAF‐associated resistance mechanism.

## 1 INTRODUCTION

Cancer does not exist in isolation within the body, rather it is encompassed by the tumor microenvironment (TME). TME consists of diverse cell types, including tumor stroma, immune cells, and stromal cells. These cells influence cancer cells’ growth and elimination by transmitting direct or indirect signals between one another.^1^

Cancer‐associated fibroblasts (CAFs) are crucial as key components within the TME. Normal fibroblasts transform into CAFs upon interaction with cancer cells. CAFs exhibit distinct characteristics from normal fibroblasts and are recognized for their ability to enhance cancer cell proliferation, invasion, and metastasis.^2^, ^3^ They achieve this by remodeling cellular substrates, secreting a variety of cytokines, and stimulating vascular production.^4^, ^5^, ^6^, ^7^ The resistance against chemo‐ and radiotherapies is multifaceted and influenced by numerous factors, with CAF being one of several contributors such as tumor cell‐intrinsic mechanisms, the presence of other stromal cells, and the tumor microenvironment as a whole.^8^, ^9^, ^10^, ^11^, ^12^ Given the significant impact of CAF on the behavior of cancer cells, CAF‐targeted treatments are expected to be a major tool for overcoming cancer cell resistance to various therapies and improving treatment performance.^13^ Therefore, many studies have investigated the interactions between CAFs and cancer cells.

One of the challenges in CAF research is lack of a stable and continuous source. Currently, CAFs are isolated directly from a patients' tumor tissue. There is a risk of CAF properties diminishing when isolated from tumor tissues and subjected to subsequent culture steps.^14^, ^15^ As a result, numerous studies have been conducted on in vitro fibroblast‐tumor co‐culture platforms to induce the transformation of normal fibroblasts into CAFs.^16^, ^17^ In most cases, the two cell types were co‐cultured in the same culture dish (direct co‐culture).^18^, ^19^ Cancer cells secrete various signaling molecules, such as growth factors, cytokines, and chemokines, which can activate nearby fibroblasts. These secreted factors induce phenotypic changes in fibroblasts, causing them to acquire CAF‐like characteristics. However, separating fibroblasts and cancer cells following direct co‐culture has limitation. Additionally, studies are underway to induce CAF traits by indirectly co‐culturing normal fibroblasts and tumor cells, allowing for the exchange of paracrine signals.^20^, ^21^ However, the expression of alpha‐smooth muscle actin (α‐SMA), a marker of CAF, is not adequately observed in these approaches due to lack of direct cell–cell interaction. Furthermore, most studies have been confined to two‐dimensional (2D) environments, which imposes a significant limitation in that they cannot replicate a comparable three‐dimensional (3D) environment within the human body.

In this study, we developed a 3D in vitro bilayer hydrogel‐based co‐culture system of fibroblasts and cancer cells to investigate the behavior and transformation of fibroblasts into CAFs in a physiologically relevant and spatially complex environment. In the bilayer system, the upper and lower layer consisted of polyethylene glycol (PEG)/collagen hydrogels entrapping fibroblasts and alginate hydrogels entrapping pancreatic cancer cells (PANC‐1), respectively. After confirming the cytocompatibility of these hydrogels with each cell type, the transformation of normal fibroblasts into CAF was monitored by observing morphological changes in the fibroblasts and the mRNA expression levels of CAF‐related markers in the co‐cultured fibroblasts. After co‐culturing, proteomic analysis was performed using the secretome from CAF to further characterize the transformed CAF. Finally, the effects of CAF on tumor resistance to radio and chemotherapies were investigated.

## 2 MATERIALS AND METHODS

### 2.1 Materials

4‐arm‐PEG‐succinimidyl glutarate (PEG‐NHS, 10 K), Triton X‐100, bovine serum albumin (BSA), collagenase, 2‐[(2‐hydroxy‐1,1‐bis(hydroxymethyl)ethyl)amino]ethanesulfonic acid (TES), sodium alginate, and calcium chloride were purchased from Sigma Aldrich (Milwaukee, WI, USA). Phosphate‐buffered saline (PBS, pH 7.4), Dulbecco's phosphate‐buffered saline (DPBS), and penicillin/streptomycin (P/S) were purchased from Gibco (Waltham, MA, USA). Fetal bovine serum (FBS), and 4,6‐diamidino‐2‐phenylindole dihydrochloride (DAPI) were purchased from Thermo Fisher Scientific (Waltham, MA, USA). Sodium hydroxide (NaOH), and sodium chloride (NaCl) were purchased from Duksan Pure Chemicals (Seoul, Korea). Pepsin soluble collagen (6 mg/mL) in 0.01 M HCl (6 mg/mL collagen solution) was purchased from Collagen Solutions (Eden Prairie, MN, USA). Cell counting kit‐8 (CCK‐8) was purchased from Dojindo (Kumamoto, Japan). Paraformaldehyde (4%) was purchased from T&I Biotechnology (Seoul, South Korea). SPLInsert™ Hanging was purchased from SPL Life Sciences (Gyeonggi‐do, South Korea).

### 2.2 Preparation of alginate and collagen/PEG hydrogels

Sodium alginate was dissolved in distilled water to prepare a 1 wt% solution, and a 25 mM calcium chloride solution was used as the cross‐linking agent. To produce alginate gel, a filter paper was soaked in the calcium chloride solution, and silicon molds with a hole diameter of 1.4 cm were placed above the filter paper. The silicon molds were fabricated with a uniform thickness of 1 mm. Each mold was filled with 200 μL of alginate solution and a filter paper soaked with calcium chloride solution was placed at the bottom of molds. An equal volume of calcium chloride solution was added to the mold, and the gelation process took 20–30 min at room temperature. After gelation, the filter papers and silicon molds were removed, and the remaining solution was aspirated.

The collagen/PEG hydrogel was fabricated following a previous study.^22^ Briefly, 1 N NaOH, deionized water, and 10x PBS were mixed at a ratio of 3:57:20 to prepare a neutralization solution. Then, a 6 mg/mL collagen solution was mixed with the neutralization solution at a 17:5 ratio. The neutralized collagen solution was kept below 4°C until use. Neutralized collagen was conjugated to 4‐arm PEG SG NHS ester via NHS chemistry to react with the primary amines of the collagen. First, PEG was dissolved in 1x PBS at a concentration of 100 mg/mL, and then 2 μL of this solution was combined with 1 mL of neutralized collagen. A mixture of PEG and collagen was then incubated at 37°C for 30 min to ensure complete gelation.

### 2.3 Cell culture

NIH/3 T3 fibroblasts were obtained from the American Type Culture Collection (ATCC, Manassas, VA, USA). PANC‐1, a pancreatic cancer cell line obtained from the Korean Cell Line Bank (Seoul, South Korea), was chosen as a model cancer cell line. NIH/3 T3 and PANC‐1 were cultured in high‐glucose DMEM supplemented with 10% FBS and 1% P/S. All cell cultures were maintained in a 5% CO_2_ incubator at 37°C and media were changed every 2–3 days.

### 2.4 2D and 3D co‐culture

For 2D co‐culture, co‐culture systems were established through 6‐well plate and 0.4 μm pore size transwell inserts. PANC‐1 cells were seeded in the Transwell inserts at a density of 1 × 10^4^ cells/well, and NIH/3 T3 cells at a density of 1 × 10^4^ cells/well in 6‐well plates. Cells were cultured in the high‐glucose DMEM supplemented with 10% FBS and 1% P/S. For 3D cell encapsulation, all the hydrogel precursor solutions were sterilized under UV light exposure. PANC‐1 cells were mixed with alginate solution, and NIH/3 T3 cells were mixed with a combination of PEG and collagen solutions. These precursor solutions were carefully pipetted several times to ensure uniform distribution of cells within the hydrogels. Then, the cell‐encapsulating hydrogels were fabricated via the polymerization steps mentioned in Section 2.2. The 3D co‐culture system of fibroblasts and cancer cells consisted of bi‐layer structures of collagen/PEG and alginate hydrogels. First, alginate hydrogels containing PANC‐1 cells were prepared and transferred to each well of a 48‐well plate. Then, 400 μL of the PEG/collagen precursor solutions containing NIH/3 T3 were added over the alginate hydrogel in each well. The samples were then incubated at 37°C for 30 min to ensure gelation. For the preparation of PANC‐1 conditioned media (CM), PANC‐1 cells were encapsulated in alginate hydrogel. After 24 h, the hydrogels encapsulating PANC‐1 cells were washed with PBS three times, and then fresh media without FBS were added. The cells were cultured for 72 h, and then the CM were collected. The obtained CM were centrifuged at 1001 rcf for 15 min, filtered with 0.22 μm filters, aliquoted, and then stored at −20°C. The CM were supplemented with 10% FBS prior to usage. The CM incubated with PEG/collagen hydrogels encapsulating NIH/3 T3 cells were changed on day 4.

### 2.5 Cell viability and proliferation assay

Cell viability was investigated using two different methods: CCK‐8 and fluorescence live/dead assays. First, fluorescence live/dead cells were used to obtain cell viability results qualitatively. The live/dead assay solution was added to the 3D culture model, and 3D culture model was incubated for 2 h. After washing twice with 1x DPBS, the cells were observed under a confocal microscope (LSM980; Carl Zeiss Inc., Thornwood, NY, USA). The proliferation of fibroblasts and tumor cells was determined using CCK‐8 assay. For 2D cell culture, NIH/3 T3 cells were seeded at a density of 7.5 × 10^4^ cells/well in a 6‐well plate, while PANC‐1 cells were seeded at a density of 1 × 10^5^ cells/well in a 12‐well plate. For 3D cell culture, NIH/3 T3 cells were encapsulated at a density of 4 × 10^5^ cells/well and PANC‐1 cells at a density of 1 × 10^5^ cells/well in 48‐well plates. Each sample was incubated for 1, 4, or 7 days. In all CCK‐8 assay experiments, the medium was replaced with a phenol red‐free and serum‐free medium. The CCK‐8 reagent was added to each well at a ratio of 10% of the medium volume and incubated for 1–3 h. The absorbance was measured at 450 nm using a microplate reader (SpectraMax ABS Plus, Molecular Devices, CA, USA).

### 2.6 Cell morphology

The morphology of the cells encapsulated within the hydrogels was assessed using DAPI/phalloidin staining. For DAPI/phalloidin staining, hydrogels encapsulating cells were fixed in 4% paraformaldehyde for 20 min. Then, they were permeabilized with 0.1% Triton X‐100 solution for 1 h and blocked with 1% BSA for 1 h in an incubator. The samples were then incubated with rhodamine phalloidin for 1.5 h at room temperature. The nuclei were counterstained with DAPI for 5 min at room temperature. The hydrogels were washed twice with DPBS at each step. After staining, the cells were imaged using a confocal microscope (LSM980; Carl Zeiss Inc., Thornwood, NY, USA). A 2D culture model was generated using a similar procedure. Quantitative analysis (cell area and roundness) was performed using ImageJ, an open‐source image analysis software. The roundness of cells was calculated using the formula 4 × π × Area/(π × major axis^2^).

### 2.7 Cell retrieval

On day 7 post‐cell encapsulation, the cell‐containing collagen/PEG hydrogel was degraded by applying a 1–2 mg/mL collagenase working solution. The collagenase solution was prepared by mixing complete medium in a 1:1 volume ratio and adding it to the hydrogel. The mixture was incubated in a shaking heat block at 37°C for 20 min. Once the hydrogel was fully digested, the degraded solution was isolated through a 40‐μm cell strainer to collect cells, which were then centrifuged at 1300 rpm for 3 min and rinsed twice with 1x DPBS. The cytotoxicity of collagenase against NIH/3 T3 fibroblasts was evaluated using the CCK‐8 assay. Briefly, the NIH/3 T3 cells were seeded at a density of 1 × 10^4^ cells/well in 48‐well plates and incubated overnight. After reaching confluence, the cells were treated with the collagenase working solution for 24–72 h and then, underwent CCK assay.

### 2.8 Quantitative real‐time polymerase chain reaction (qRT‐PCR)

After co‐culturing for 7 days, collagen/PEG hydrogels containing NIH/3 T3 cells were separated from the lower layer, which was then degraded by collagenase to retrieve the encapsulated cells. RNA was extracted from the retrieved cells. Whole RNA in fibroblasts was isolated using ReliaPrep™ RNA Miniprep Systems (Promega, Madison, USA). Complementary DNA (cDNA) was subsequently synthesized from 1 μg total RNA utilizing AccuPower® RT PreMix (Bioneer, Daejeon, Korea), in accordance with the manufacturer's instructions. Thereafter, cDNA was amplified by Quantitative real‐time PCR using Power SYBR™ Green PCR Master Mix (Applied Biosystems, Warrington, UK). Glyceraldehyde‐3 phosphate dehydrogenase (GAPDH) expression was used as an internal control for normalization. The primer sequences used for qRT‐PCR are listed in Table 1.

**TABLE 1:** Sequences of primers used for qRT‐PCR.

Primer	Forward sequence (5′‐3′)	Reverse sequence (5′‐3′)
α‐SMA (Acta2)	GTCCCAGACATCAGGGAGTAA	TCGGATACTTCAGCGTCAGGA
FAP	GTCACCTGATCGGCAATTTGT	CCCCATTCTGAAGGTCGTAGAT
FSP1 (S100a4)	TCCACAAATACTCAGGCAAAGAG	GCAGCTCCCTGGTCAGTAG
CSPG4	GGGCTGTGCTGTCTGTTGA	TGATTCCCTTCAGGTAAGGCA
CXCL12	TTCTTCGAGAGCCACATCGC	TTTCGGGTCAATGCACACTT
HGF	ATGTGGGGGACCAAACTTCTG	GGATGGCGACATGAAGCAG
GAPDH	AGGTCGGTGTGAACGGATTTG	TGTAGACCATGTAGTTGAGGTCA

Abbreviations: CSPG4, chondroitin sulfate proteoglycan 4; CXCL12, C‐X‐C motif chemokine ligand 12; FAP, fibroblast activation protein; FSP‐1, fibroblast stimulating protein‐1; GAPDH, glyceraldehyde‐3 phosphate dehydrogenase; HGF, hepatocyte growth factor; qRT‐PCR, quantitative real‐time polymerase chain reaction; α‐SMA, alpha‐smooth muscle actin.

### 2.9 Study of radio and drug resistances of co‐cultured cancer cells

NIH/3 T3 fibroblasts and PANC‐1 cells were co‐cultured in the hydrogel for 7 days before exposure to ionizing radiation and drug treatment. Subsequently, the hydrogels were subjected to irradiation and drug treatment at different doses and incubated for an additional 3 days. The survival rate of PANC‐1 cells was measured using a CCK‐8 assay after separating the upper layer encapsulating fibroblasts. To investigate the effect of co‐culture with CAF on the radioresistance of cancer cells, co‐culture hydrogel system encapsulating NIH/3 T3 and PANC‐1 cells was exposed to various doses of radiation, including 2, 4, 6, and 8 Gy, using X‐rad 320 (Precision X‐Ray, North Branford, CT, USA). The radiation fraction was operated at 300 kV, 12.5 mA with 1.0 mm Al filtration and a distance of approximately 69 cm from the object being irradiated. Appropriate radiation dose was determined through pre‐irradiation tests, which observed that the survival rate fell below 50% at the 4 Gy irradiation dose in the mono‐cultured group. Anti‐cancer drug resistance of PANC‐1 cells was investigated by treating mono‐ or co‐cultured PANC‐1 cells with gemcitabine. The gemcitabine concentrations used in these experiments ranged from 0.1 to 100 μM. This concentration range was determined based on reference papers,^23^, ^24^, ^25^ aiming for a concentration that would yield a survival rate of 50% or lower when treated with gemcitabine.

### 2.10 Liquid Chromatography with tandem mass spectrometry (LC‐MS/MS) Analysis

After 7 days of co‐culture, the collagen/PEG hydrogels were separated from the co‐culture system and cultured in serum‐ and phenol‐red‐free media for 48 h. The conditioned media were used immediately or stored at −80°C. Protein concentrations in the samples were determined using a Bradford assay. Each sample containing 100 μg of protein was serially subjected to in‐solution digestion and desalting. Disulfide bonds in the samples were reduced by 1/20 sample volume of 500 mM DTT in 50 mM ABC for 30 min incubation at 60°C and alkylated by 1/20 sample volume of 500 mM IAA in 50 mM for 30 min reaction at RT in the dark. 2 μg of trypsin protease in 50 mM ABC was added to the sample adjusting the final sample volume to 200 μL followed by incubation at 37°C for 16–18 h for protein digestion. Digested samples were desalted on QuikPrep® Macro SpinColumns™ C18 (Harvard Apparatus, Holliston, MA, USA) for reverse‐phase chromatography and eluted with 80% ACN in 0.1% TFA. Eluted proteins were dried and resuspended in 0.1% formic acid in water. Then, 1–2 μg of protein from the resuspended sample was injected into a Q‐Exactive HF‐X column (Thermo Fisher Scientific, Waltham, MA, USA) for liquid chromatography‐mass spectrometry (LC‐MS). Thermo Scientific™ Proteome Discoverer™ Software 3.0 was used for the identification of proteins from LC‐MS data. The exported data were normalized to the sum of each abundance value, and differential protein expression was assessed using a two‐sided *t*‐test with a significance threshold of 0.05. Among the up‐regulated proteins, CAF‐related proteins were investigated using the Data resource of Cancer‐Associated Fibroblast (http://caf.zbiolab.cn/index.php).

### 2.11 Statistical Analysis

The results were presented as means ± standard error of the mean (SEM) from at least three independent experiments. Statistical analyses were performed using GraphPad Prism 8.4.2 (GraphPad Software, San Diego, CA, USA) with Student's t‐test or analysis of variance (ANOVA) within the same group and two‐way ANOVA across different groups, followed by Tukey's multiple comparison test. A *p*‐value of less than 0.05 was considered statistically significant (*p < 0.05, **p < 0.005, and ***p < 0.001).

## 3 RESULTS

### 3.1 Fabrication of 3D co‐culture platform

**FIGURE 1:**
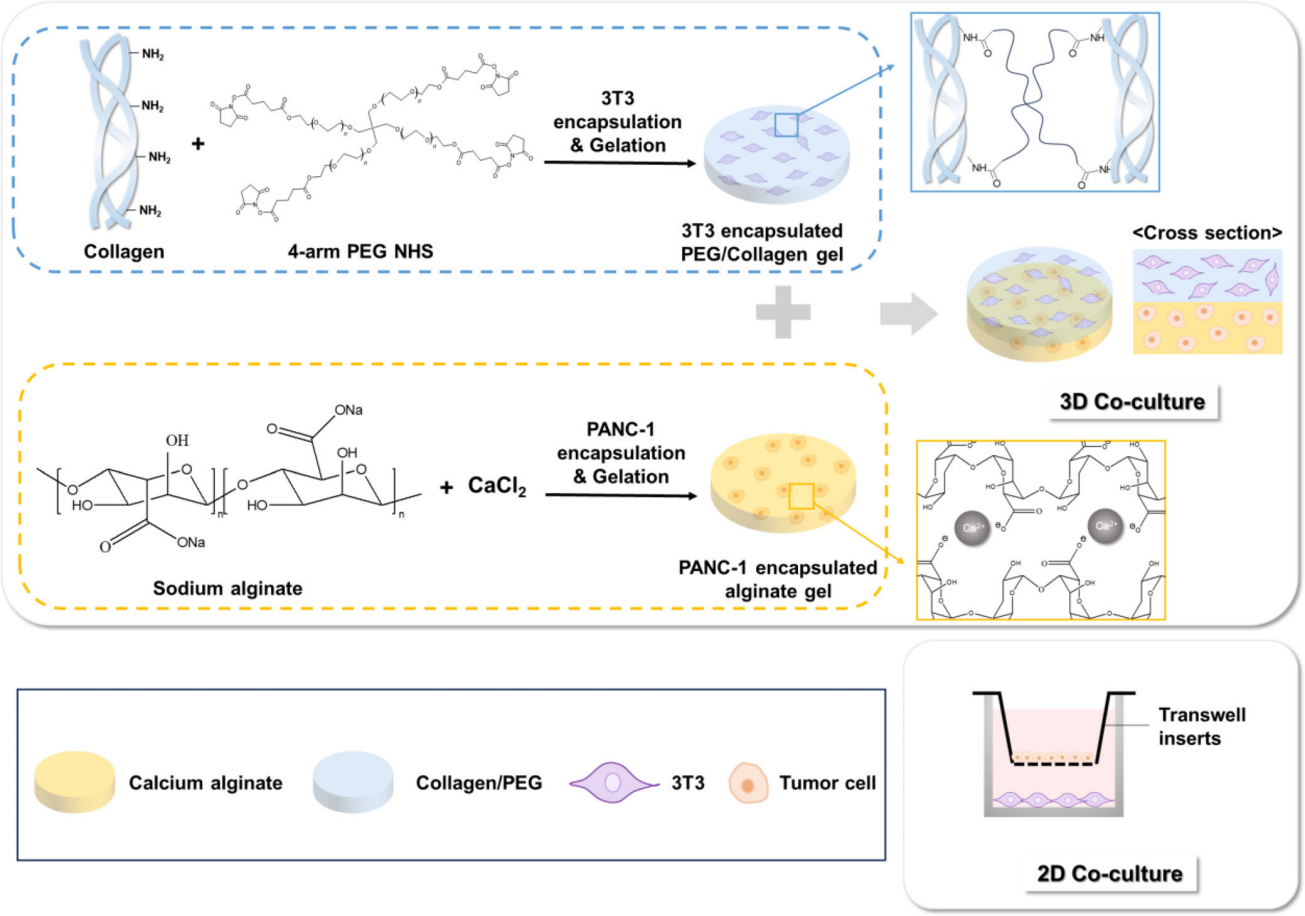
Schematic illustration of preparing three‐dimensional (3D) and two‐dimensional (2D) co‐culture platform.

The 3D co‐culture platform comprised dual hydrogel layers, wherein the upper layer consisted of a collagen/PEG hydrogel for NIH/3 T3 fibroblast culture and the lower layer was composed of an alginate hydrogel for PANC‐1 cell culture (Figure 1). For the collagen/PEG hydrogel, 4‐arm PEG functionalized with NHS was used to crosslink the collagen.^22^ Alginate hydrogels were prepared using calcium chloride as the crosslinking agent. For comparison, a 2D co‐culture system was established using a Transwell insert.

In the resultant bilayer hydrogel, the adhesion between different hydrogels encapsulating NIH/3 T3 and PANC‐1 cells was strong enough to maintain a bilayer structure throughout the culture period but weak enough to be separated by applying physical forces (Movie S1).

First, rheological properties were analyzed using a rheometer to confirm the gelation and investigate the mechanical properties of each hydrogel. According to Figure S1, complete gelation of both hydrogels was verified by the fact that the storage moduli (G′) of both hydrogels were higher than their loss moduli (G′′). The alginate hydrogel exhibited higher rheological strength than the collagen/PEG hydrogel.

**FIGURE 2:**
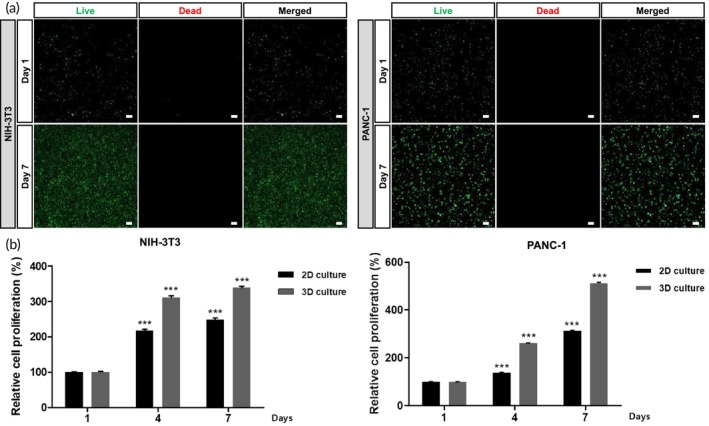
The cytocompatibility of hydrogels. (a) Live/dead fluorescence images of NIH/3T3 encapsulated in collagen/polyethylene glycol (PEG) hydrogel as well as pancreatic cancer cells (PANC‐1) encapsulated in alginate hydrogel. (b) The proliferation of NIH/3T3 in collagen/PEG hydrogel and PANC‐1 in alginate hydrogel. Scale bars correspond to 100 μm. Statistical analysis was performed by comparing with day1. ***p  <  0.001.

The viability of the cells within the hydrogels was evaluated using a live/dead assay for 7 days, as illustrated in Figure 2a. Most of the encapsulated cells emitted green fluorescence and the number of cells increased, indicating that NIH/3 T3 and PANC‐1 cells remained alive and proliferated for 7 days in both hydrogels. The proliferative capacity of the cells within the hydrogels was further investigated using a CCK‐8 assay (Figure 2b). The cells exhibited substantial proliferation on both hydrogels. The proliferation rate of 3D encapsulated cells was significantly higher than of the 2D‐cultured cells on conventional culture dishes. We also continuously monitored the location of NIH/3 T3 and PANC‐1 cells during the 7 days of culture period through cell labeling and confirmed that NIH/3 T3 and PANC‐1 cells remained well separated in each hydrogel layers without migrating to different layers (Figure S2).

An important feature of the proposed bilayer system is that cells encapsulated within the collagen/PEG hydrogel can be retrieved by collagenase treatment. Since collagenase degrades only the collagen/PEG hydrogel without affecting the alginate hydrogel, CAFs transformed from NIH/3 T3 cells could be selectively collected. The cytotoxicity of collagenase against NIH/3 T3 cells was evaluated using the CCK‐8 assay (Figure S3a). 2D mono‐cultured NIH/3 T3 cells treated with collagenase working solution showed good proliferation rate until day 2. Considering that the exposure time to the collagenase solution in this study was only 20 min, retrieval of cells from the collagen/PEG hydrogel did not affect cell viability. The viability of the retrieved NIH/3 T3 cells was also verified using a live/dead assay (Figure S3b). Here, NIH/3 T3 cells encapsulated in a collagen/PEG hydrogel with and without co‐culture with PANC‐1 cells were monitored. Figure S3b shows the resultant fluorescence images obtained 3 days after retrieval, which confirmed that the retrieved cells grew well and remained alive in both cases. However, NIH/3 T3 cells co‐cultured with PANC‐1 exhibited better proliferation than mono‐cultured cells.

### 3.2 Effect of co‐culture on NIH/3 T3 and PANC‐1

All co‐culture experiments were conducted for 7 days to provide sufficient time for NIH/3 T3 and PANC‐1 cells to establish significant interactions within the co‐culture platform.

#### 3.2.1 Morphology changes of NIH/3 T3 cells

**FIGURE 3:**
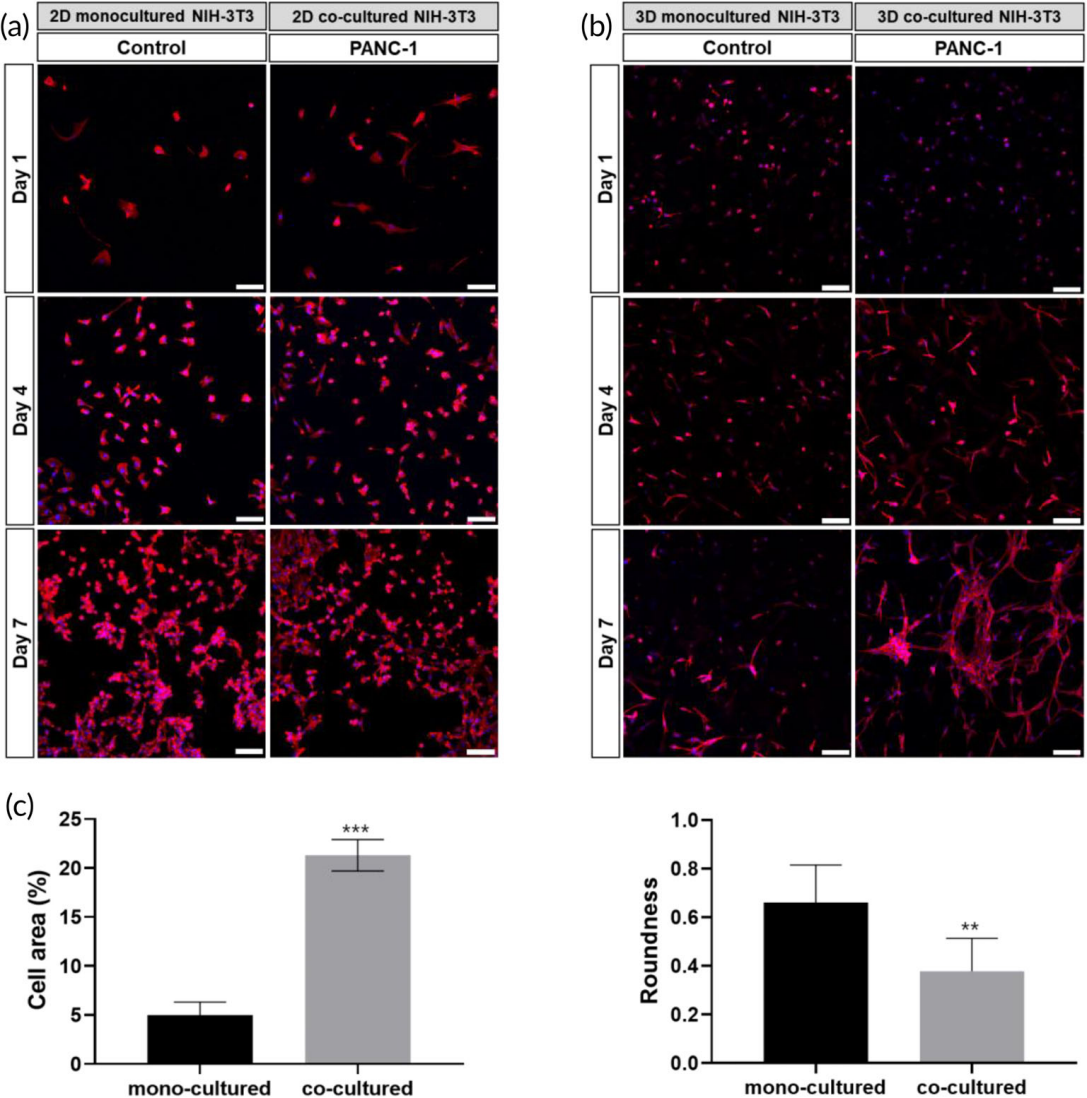
4,6‐diamidino‐2‐phenylindole dihydrochloride (DAPI)/Phalloidin staining showing the morphology of mono‐ and co‐cultured NIH/3T3 at (a) two‐dimensional (2D) and (b) three‐dimensional (3D) culture conditions. Scale bars correspond to 100 μm. (c) Cell area (%) and roundness of mono‐ and co‐cultured 3T3/NIH for 7 days under 3D culture conditions. PANC‐1, pancreatic cancer cells. **p < 0.005; ***p <  0.001.

The fluorescence images (Figure 3a,b) show the time‐dependent morphological changes of NIH/3 T3 cells cultured in the 2D and 3D co‐culture platforms. Mono‐cultured NIH/3 T3 cells exhibited a thin and small spindle morphology throughout the culture period. In contrast, NIH/3 T3 cells co‐cultured with PANC‐1 cells exhibited a large and plump spindle shape, which is characteristic of CAFs. In addition, morphological differences between mono‐ and co‐cultured NIH/3 T3 cells were more prominent in the 3D microenvironment. Figure 3c demonstrates an increase of cell area and a decrease of roundness for 3D co‐cultured NIH/3 T3 cells compared with 3D mono‐cultured NIH/3 T3 cells, corroborating our observations of morphological transformation.

#### 3.2.2 Proliferation of PANC‐1

The influence of the NIH/3 T3 co‐culture on PANC‐1 proliferation was investigated using the CCK‐8 assay (Figure S4). The upper collagen/PEG hydrogels were removed after 1, 4, and 7 days of culture, and the CCK‐8 assay was performed for the PANC‐1 cells encapsulated within the alginate hydrogels. Co‐cultured PANC‐1 cells showed a difference in proliferation rate compared to mono‐cultured cells on day 4. Moreover, on day 7, the degree of increase in the co‐cultured group was significantly higher than that in the mono‐culture group.

### 3.3 mRNA expression levels of CAF‐related markers

**FIGURE 4:**
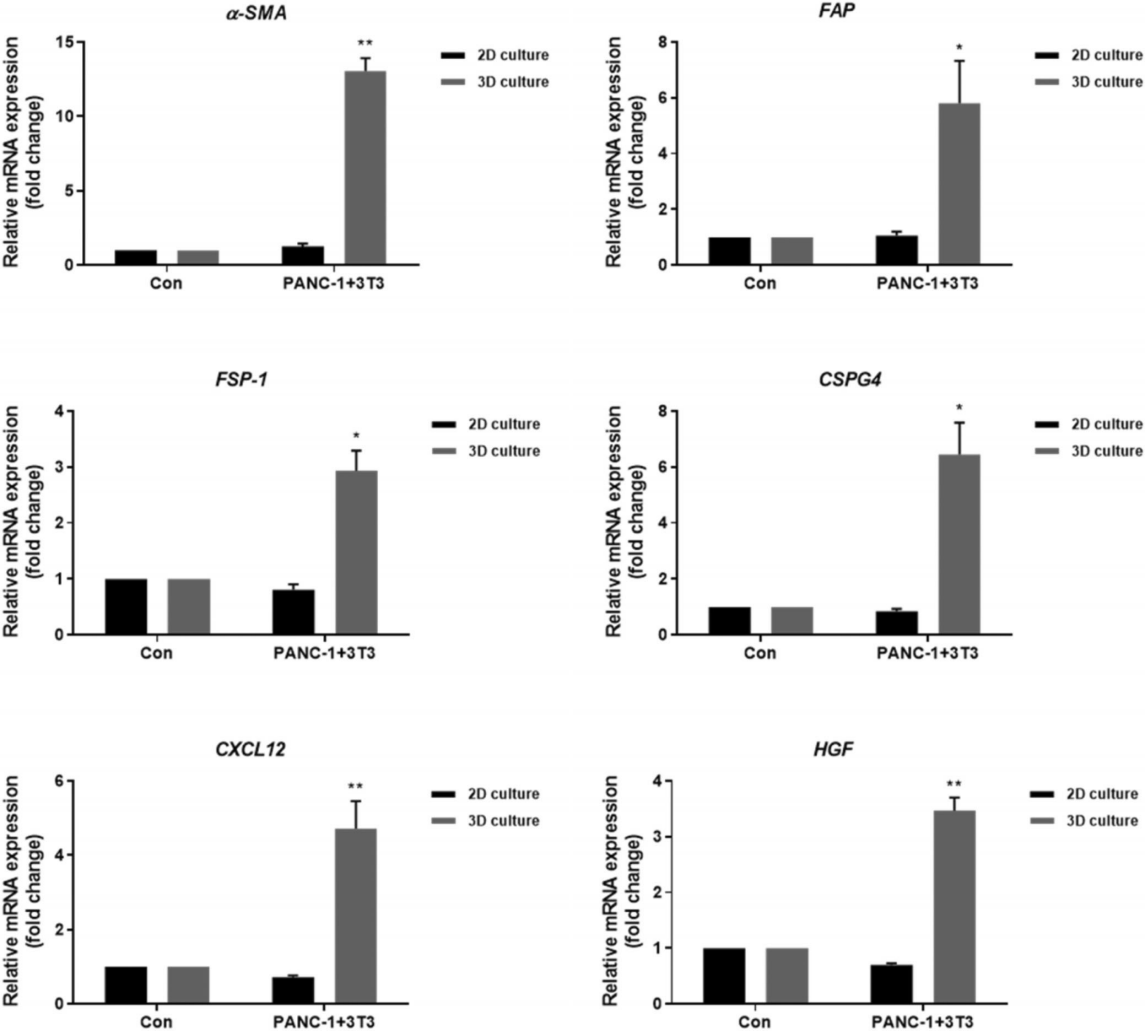
Verification of mRNA expression levels of cancer‐associated fibroblast (CAF)‐related markers in NIH/3T3 cells co‐cultured with pancreatic cancer cells (PANC‐1). alpha‐smooth muscle actin (α‐SMA), fibroblast activation protein (FAP), fibroblast stimulating protein‐1 (FSP‐1), chondroitin sulfate proteoglycan 4 (CSPG4), C‐X‐C motif chemokine ligand 12 (CXCL12), and hepatocyte growth factor (HGF) mRNA levels were determined by real‐time polymerase chain reaction (RT‐PCR). *p < 0.05 and **p < 0.005.

No unique markers were identified that distinguished CAFs from normal fibroblasts (NFs). Although a CAF marker with absolute specificity has not been identified, several well‐established indicators of CAFs, such as α‐smooth muscle actin (α‐SMA), fibroblast activation protein (FAP) and fibroblast stimulating protein‐1 (FSP‐1), exist.^26^, ^27^ Additionally, CAF‐secreted factors, including the C‐X‐C motif chemokine ligand 12 (CXCL12), chondroitin sulfate proteoglycan 4 (CSPG4), and hepatocyte growth factor (HGF), are upregulated upon the activation of NFs into CAFs.^28^, ^29^, ^30^ In this study, the differentiation of NFs into CAFs via co‐culture with cancer cells was confirmed by quantifying the mRNA expression levels of α‐SMA, FAP, FSP‐1, CSPG4, CXCL12, and HGF using qRT‐PCR. In 3D culture environment, the NIH/3 T3 cells co‐cultured with PANC‐1 cells significantly upregulated the mRNA levels of α‐SMA, FAP, FSP‐1, CSPG4, CXCL12 and HGF by 13.0‐, 5.8‐, 2.9‐, 6.4‐, 4.7‐ and 3.4‐fold, respectively compared with mono‐cultured NIH/3 T3 cells (controls) (Figure 4). Significant upregulation of these markers observed in 3D co‐culture system was not observed in the 2D indirect co‐culture system. Furthermore, Figure S5 demonstrates that while conditioned media can induce some activation of 3 T3 cells into CAFs, there was no significant difference from the control. Direct co‐culture with 3D PANC‐1 cells resulted in significantly higher expression levels of α‐SMA and FSP‐1 than conditioned media. This highlights the importance of dynamic interactions in a 3D environment for accurately replicating the tumor microenvironment and effectively activating fibroblasts into CAFs.

### 3.4 Proteomic analysis

**FIGURE 5:**
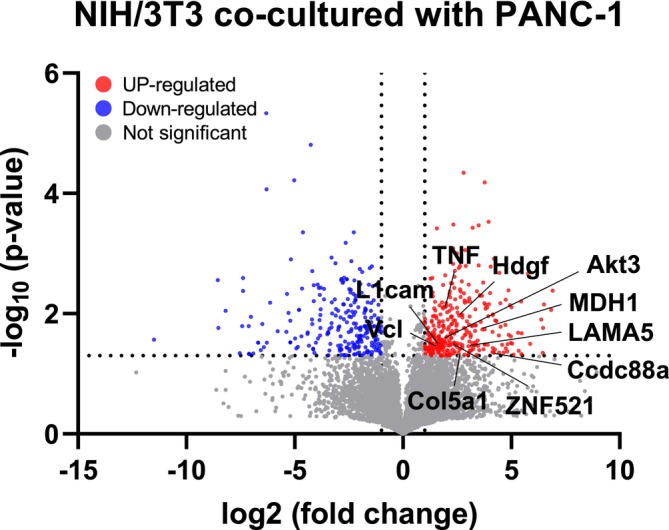
Volcano plot of differentially expressed proteins (DEPs); cancer‐associated fibroblast (CAF)‐related proteins that showed up‐regulation are marked in the plot.

A total of 22,447 proteins were successfully identified using liquid chromatography‐mass spectrometry (LC–MS). Among these, 590 proteins exhibited a *p*‐value <0.05, indicating statistical significance (Figure S6a). The exploration of differentially expressed proteins (DEPs) involved scrutinizing how these 590 proteins, identified as statistically significant, were differentially regulated in the context of 3D co‐cultured NIH/3 T3 cells compared with mono‐cultured NIH/3 T3 cells (control). Visualization of this information was achieved using heatmap images, as shown in Figure S6b. Specifically, upregulated proteins were systematically listed based on their fold‐change (sample/control) values (Table S1). Subsequently, selective identification was performed using the Cancer‐Associated Fibroblast Marker Database (http://caf.zbiolab.cn/index.php) to isolate proteins associated with CAFs that were up‐regulated in the samples. Figure 5 shows the volcano plot of DEPs; among the up‐regulated proteins, CAF‐related proteins selected through this process are marked. Table 2 shows a list of the up‐regulated CAF‐related proteins.

**TABLE 2:** Up‐regulated proteins related to CAF in 3D co‐culture compared with 3D mono‐culture platforms.

Gene name	Protein name	CAF type	Fold change	*p*‐value
Ccdc88a	Girdin	Responsor	18.08589	0.043688
MDH1	Malate dehydrogenase, cytoplasmic	Secretion; responsor	12.6592	0.018339
LAMA5	Laminin subunit alpha‐5	Secretion; responsor	8.123264	0.035065
Hdgf	Hepatoma‐derived growth factor	Responsor	6.027185	0.010833
Col5a1	Collagen alpha‐1(V) chain	Secretion; responsor	5.881912	0.044671
ZNF521	Zinc finger protein 521	Responsor	5.244096	0.032349
Akt3	RAC‐gamma serine/threonine‐protein kinase	Responsor	3.689334	0.027048
TNF	Tumor necrosis factor	Secretion; regulator; responsor	3.562308	0.007927
L1cam	Neural cell adhesion molecule L1	Regulator	3.098636	0.032535
Vcl	Vinculin	Secretion	3.064191	0.037472

Abbreviations: 3D, three‐dimensional; CAF, cancer‐associated fibroblast.

Proteins listed in the table are known CAF‐related proteins or those associated with promoting cancer proliferation. Girdin, an Akt phosphorylation enhancer, and Akt3 are involved in actin organization and cell motility, affecting cancer cell invasion, progression, and angiogenesis.^31^, ^32^ Importantly, both proteins were up‐regulated in the samples by 18‐ and 3‐fold, respectively. Malate dehydrogenase, an enzyme involved in NAD^+^ regeneration during glycolysis, is known to influence cancer metabolism.^33^ It also exhibited a relatively high up‐regulation (approximately 13‐fold). Laminin and collagen alpha‐1(V) chains are associated with extracellular matrix (ECM), which is known to be involved in cancer cell metastasis,^34^, ^35^ and also exhibited 8‐ and 6‐folds up‐regulation, respectively. Similarly, hepatoma‐derived growth factor showing 6‐fold upregulation has been implicated in cancer cell growth and metastasis and proposed as a novel prognostic factor for pancreatic cancer.^36^ Additionally, vinculin, zinc finger protein 521, and neural cell adhesion molecule L1 are known to play roles in cancer development, proliferation, and metastasis,^37^, ^38^, ^39^ and underscored significant elevation in expression levels as well.

### 3.5 Effect of co‐culture with NIH/3 T3 on radio‐ and chemo‐resistance of PANC‐1

**FIGURE 6:**
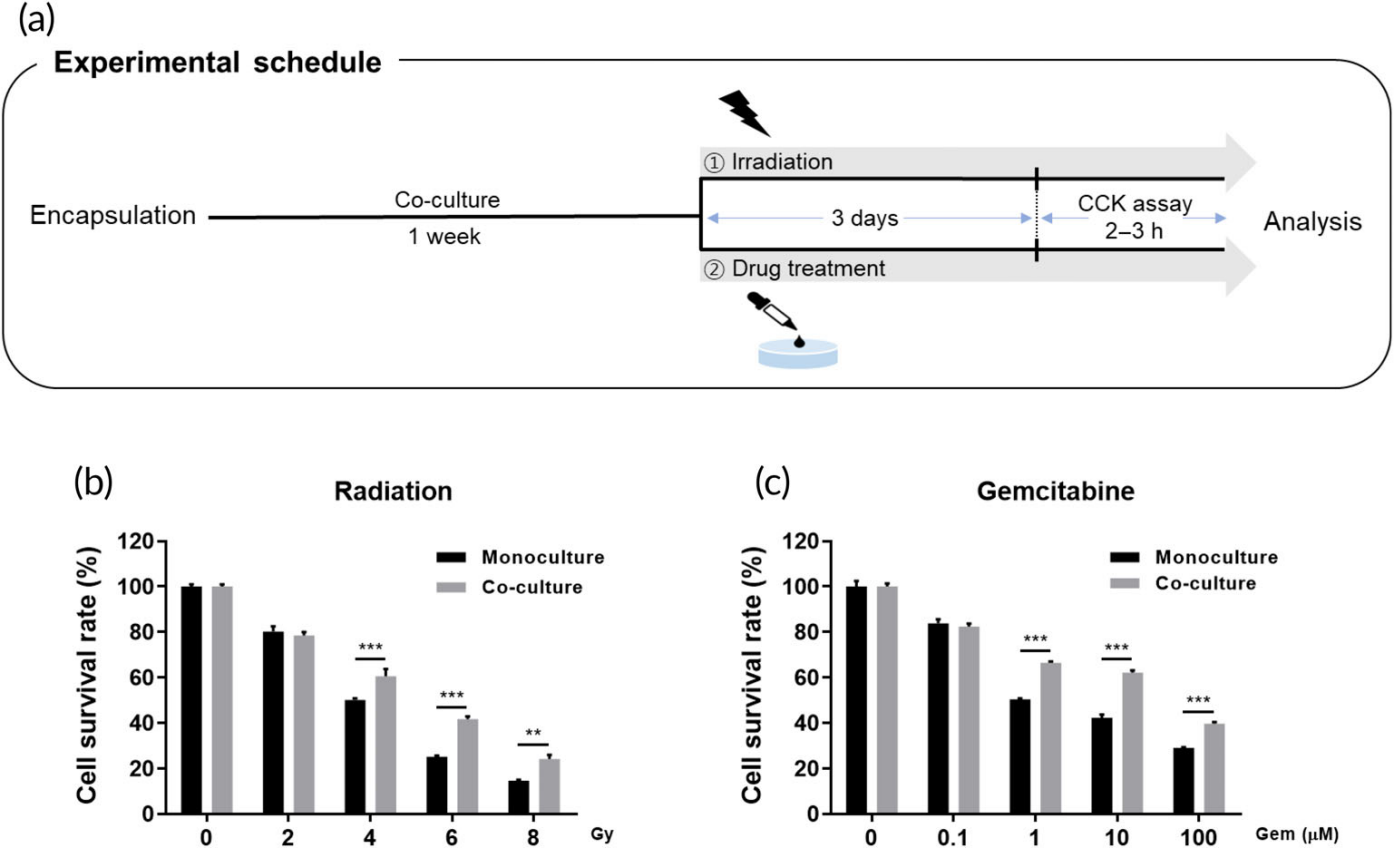
Radio‐ and chemoresistance of tumor cells induced by co‐culture with NIH/3T3 cells. (a) Schematic diagram of experimental schedule. Radio‐ and chemoresistance of tumor cells was investigated in three‐dimensional (3D) culture environments. (b) Cell counting kit‐8 (CCK‐8) assays were employed to assess the effect of varying doses of x‐ray irradiation on pancreatic cancer cells (PANC‐1) over a 72‐h exposure period. (c) Additionally, the survival rate of PANC‐1 cells after a 72‐h treatment with gemcitabine was evaluated using the CCK‐8 assay. **p < 0.005; ***p < 0.001.

Several studies have reported that CAFs promote growth and radioresistance of cancer cells, both in vivo and in vitro.^10^, ^40^ Here, we utilized our proposed co‐culture platforms to investigate the contribution of co‐cultured CAFs to the radioresistance of pancreatic cancer cells, as shown in Figure 6a. When co‐cultured PANC‐1 cells were subjected to radiation ranging from 0 ~ 8 Gy after 7 days of co‐culture, the survival rate of PANC‐1 cells exhibited a remarkable increase compared to mono‐culture conditions. This effect was particularly pronounced in the 4 ~ 8 Gy range (Figure 6b).

Since 1997, gemcitabine therapy, which is widely used in combination with radiotherapy for the treatment of pancreatic cancer, has served as the standard first‐line treatment for patients with unresectable locally advanced or metastatic disease.^41^ CAFs are potential therapeutic targets for advanced pancreatic cancer by interacting closely with tumor cells and playing an important role in drug resistance.^42^, ^43^ To mimic the drug resistance of the TME induced by CAF, we applied the proposed co‐culture system to monitor CAF‐induced drug resistance, as described in Figure 6a. Considering the cellular toxicity of anticancer drugs, PANC‐1 cells were treated with gemcitabine at concentrations of 0.1, 1, 10, and 100 μM for a duration of 3 days each. The analysis revealed that co‐cultured PANC‐1 cells exhibited notable resistance to anti‐cancer drug at concentrations of 1, 10, and 100 μM (Figure 6c), and this acquired drug resistance was statistically significant.

## 4 DISCUSSION

In in vivo, cells exist in multilayered arrangements, resulting in variations in their exposure to oxygen, nutrients, and signaling molecules based on their proximity to blood vessels. Numerous essential physiological processes such as proliferation and angiogenesis are dependent on these gradients in transport. In particular, the ECM plays a pivotal role in processes such as tumorigenesis, tumor progression, and cell migration by regulating signaling pathways through interactions with cell‐surface receptors and contact with growth factors.^44^ Therefore, establishing an environment similar to in vivo as much as possible to obtain a CAF similar to what is actually isolating in patients was crucial.

In this study, we developed an in vitro bilayer 3D co‐culture platform that simulates an in vivo environment to induce CAF activation by co‐culturing NIH/3 T3 fibroblasts and cancer cells. We selected the upper layers to consist of collagen/PEG hydrogel encapsulating NIH/3 T3 cells, and the lower layer to consist of alginate hydrogel encapsulating tumor cells. One reason is capability of selective retrieval of each cell. Here, the collagen/PEG and alginate hydrogels can be degraded by collagenase and EDTA, respectively, which allows the selective retrieval and analysis of fibroblasts and cancer cells. The ability to selectively digest one hydrogel layer (collagen/PEG) without affecting the other (alginate) enables the isolation and analysis of specific cell populations post‐co‐culture. This selective retrieval is particularly important for downstream molecular analyses, such as RNA sequencing or proteomics, where contamination with other cell types could confound results. However, this arrangement was not solely for ease of separation but was also driven by the distinct roles each material plays in mimicking different aspects of the tumor microenvironment (TME). The ECM of tumor tissue is primarily composed of collagen, thus NIH/3 T3 were encapsulated.^45^ Simple collagen, due to its weak properties, could potentially degrade or is not enough to a physically isolated environment between cells during the co‐culture period. However, by crosslinking with PEG, a more stable co‐culture platform could be maintained. Alginate is one of the most commonly used hydrogels for cell encapsulation.^46^, ^47^, ^48^ Alginate hydrogels offer a mechanically stable and biocompatible environment that can be easily tuned to match the rigidity of tumor tissues. According to rheological studies (Figure S1), alginate hydrogel exhibited a higher modulus compared to collagen/PEG hydrogel. Given that tumor tissue is typically more rigid than normal tissue, alginate hydrogel was chosen to encapsulate PANC‐1 cells.^49^ Recent studies have utilized alginate in a microbead form, taking advantage of its characteristic of low cell adhesion, to induce cancer cells to form spheroids within the beads, thereby better mimicking the in vivo environment.^50^, ^51^, ^52^ While our study did not achieve the formation of cancer cell spheroids using bulky gels without specific manipulation, alginate was selected as the material for cancer cell encapsulation, with the prospect of subsequent research to create a 3D co‐culture platform of tumor spheroids within alginate beads and fibroblasts. Both the hydrogels were cytocompatible and supported the proliferation of NIH/3 T3 and PANC‐1 cells (Figure 2). However, cell migration to different hydrogels did not occur (Figure S2), indicating that there was no direct cell–cell interaction and only indirect exchange of paracrine signals was possible between NIH/3 T3 and PANC‐1 cells.

When co‐cultured, NIH/3 T3 cells underwent changes in cell size, becoming larger and more elongated than mono‐cultured NIH/3 T3 cells, which is characteristic of CAFs (Figure 3). CAFs are large spindle‐shaped cells with well‐developed fibronectin, indented nuclei, and stress fibers.^53^ Morphological changes in NIH/3 T3 cells into CAFs when co‐cultured with PANC‐1 cells can be attributed to the signaling crosstalk between these two cell types.^27^, ^54^ For example, the secretion of transforming growth factor‐beta (TGF‐β) by cancer cells leads to the activation of CAFs and changes in their morphology. Furthermore, PANC‐1 can induce CAFs to become more contractile by upregulating the expression of contractile proteins like α‐SMA. This contractile phenotype causes CAFs to adopt a more elongated and spindle‐like morphology.^55^ In contrast, the PANC‐1 cells exhibited a significantly higher proliferation rate in the presence of NIH/3 T3 cells (Figure S4). It is well‐known that CAFs contribute to an environment that supports tumor cell proliferation through various pro‐tumorigenic effects and interactions that CAFs can have on cancer cells.^3^, ^27^ Paracrine signaling is a key factor that promotes cancer cell proliferation. For example, CAFs secrete a range of signaling molecules, including growth factors and cytokines, which can stimulate the proliferation of nearby cancer cells.^3^, ^56^ Therefore, morphological changes in NIH/3 T3 cells and enhanced proliferation rate of PANC‐1 cells support the activation of NIH/3 T3 fibroblasts via co‐culture with PANC‐1 in our 3D co‐culture platform.

In the study, we demonstrated the transformation of NFs into CAFs within a 3D hydrogel co‐culture model by examining changes in various markers associated with CAFs (Figure 4). Several markers, including α‐SMA and S100A4 protein/fibroblast‐specific protein‐1 (FSP‐1), are commonly used to identify CAFs.^26^ Moreover, local fibroblasts, which are considered a major source of CAFs, can be stimulated by autocrine/paracrine CXCL12, and CAFs can induce tumor progression through direct and indirect effects.^57^ The fibroblasts secrete growth factors such as HGF, which is an important chemotactic factor released by CAFs. HGF binds to c‐MET receptors on tumor cells, stimulating tumor invasion and growth. These CAF‐related markers contribute to the survival, proliferation, and invasiveness of cancer cells.^58^, ^59^ Considering the increased expression levels of typical CAF markers, we conclude that normal NIH/3 T3 fibroblasts acquired CAF phenotypic characteristics and promoted the malignant features of CAFs in our study model. In addition, unlike our 3D co‐culture model, NIH/3 T3 cells cultured with PANC‐1 conditioned media (CM) did not exhibit high mRNA expression of α‐SMA and FSP‐1 (Figure S5). During co‐culture, two types of cells do not have direct contact but influence each other through paracrine signaling.^60^, ^61^ For instance, paracrine signals released by cancer cells affect fibroblasts, while fibroblasts, in turn, affect the behaviors of cancer cells. When CM from one cell type were collected and added to the culture of another cell type, we would observe unilateral effects mediated solely through CM rather than mutual interactions between the two cell types. While CM experiments provide valuable information about secreted factors and their effects, they lack the spatial context and direct interaction dynamics that are crucial for understanding the full spectrum of cellular behavior within the TME.

Successful activation of NIH/3 T3 cells by 3D co‐culture was also confirmed by LC‐MS analysis as well. The DEPs showed that various CAF‐related proteins or proteins affecting cancer cell proliferation were up‐regulated in fibroblasts 3D co‐cultured with PANC‐1 cells (Figure 5 and Table 2). Girdin, Akt, Laminin, and Collagen alpha‐1(V) chain are associated with actin and ECM, facilitating cancer cell metastasis.^31^, ^32^, ^34^, ^35^ Malate dehydrogenase, hepatoma‐derived growth factor, Vinculin, Zinc finger protein 521, and neural cell adhesion molecule L1 contribute to cancer cell proliferation and growth.^33^, ^36^, ^37^, ^38^, ^39^ The up‐regulation of these proteins suggests that CAFs generated through 3D co‐culture exhibit typical CAF characteristics. These CAFs appear to release proteins that facilitate the proliferation of neighboring cancer cells, affirming their role in supporting cancer cell proliferation.

The resistance of cancer cells to irradiation or anti‐cancer drugs remains a major challenge in the treatment of various types of cancer.^62^, ^63^, ^64^ In our proposed platform, CAFs significantly enhanced the radiation resistance of PANC‐1 cells to a wide range of x‐ray absorbed doses (2–10 Gy) (Figure 6). Furthermore, co‐culturing with NIH/3 T3 cells significantly worsened the response rate of PANC‐1 cells to gemcitabine at concentrations of 1, 10, and 100 μM (Figure 6). The CAF‐related proteins identified through LC‐MS analysis corroborate the results of the radio‐ and chemoresistance experiments. Girdin, which exhibited the highest fold change, enhances the Warburg effect and chemoresistance in tumor cells through its interaction with pyruvate kinase M2 (PKM2).^65^ Akt plays a crucial role in cell survival under apoptotic stimuli, thereby promoting therapeutic resistance.^66^ Additionally, L1CAM significantly contributes to epithelial‐mesenchymal transition (EMT) and cancer‐initiating cell (CIC) formation, further enhancing resistance to chemotherapeutic drugs.^67^

Previous studies have shown that CAFs are crucial in various aspects of chemotherapy and in the survival and regrowth of irradiated cancer cells.^11^, ^68^, ^69^ However, most of these studies utilized patient‐derived CAFs, and the results were primarily demonstrated in in vivo models. We successfully replicated the results of in vivo studies using a 3D co‐culture model that mimicked the tumor microenvironment. Our in vitro model offers an easier method for generating CAFs and conducting experiments, making it readily applicable to other research endeavors. One of its major strengths lies in the research concerning CAF‐related chemo‐ and radioresistance. We anticipate that this model can be used to develop CAF‐targeted drugs to overcome radio‐ and chemotherapy resistance. Moreover, its simplicity makes it a promising tool for efficient drug screening.

Our study was designed based on prior studies that involved co‐culturing NIH/3 T3 cells with human‐origin cancer cell lines.^70^, ^71^, ^72^, ^73^ The selection of NIH/3 T3 cells was based on their well‐characterized properties and widespread use in cancer‐associated fibroblast (CAF) research. These cells provide a robust model to study fibroblast activation and the subsequent transformation into CAFs. However, we recognize that interspecies differences may influence the interactions and behavior of co‐cultured cells. To address this limitation and enhance the translational potential of our research, future studies will incorporate human‐derived fibroblast cell lines, such as human dermal fibroblasts (HDFs) or primary human pancreatic fibroblasts, alongside PANC‐1 cells. This approach will ensure that the cellular interactions and microenvironment more accurately reflect human physiological and pathological conditions. Additionally, employing human‐derived cell lines will enable us to better investigate the molecular and phenotypic changes associated with CAF activation in a human‐specific context, providing more relevant insights into the tumor microenvironment and potential therapeutic targets.

In summary, this 3D co‐culture platform successfully induced CAF activation, demonstrating its potential for studying complex interactions between fibroblasts and cancer cells in a more physiologically relevant environment.

## 5 CONCLUSION

This study presents a novel 3D in vitro co‐culture model for studying the behavior and transformation of fibroblasts into CAFs in a physiologically relevant and spatially complex environment. The developed co‐culture platform effectively induced the activation of fibroblasts into CAFs, as demonstrated by changes in morphology and up‐regulation of CAF‐related markers. Additionally, the co‐cultured cancer cells displayed increased resistance to radio‐ and chemo‐therapies, which is consistent with the pro‐tumorigenic effects of CAFs. The in vitro fibroblast‐tumor cell co‐culture platforms proposed in this study provide simple ways to obtain the CAFs, otherwise which should be obtained from real tumor microenvironment. Furthermore, this 3D co‐culture model provides a valuable tool for studying the interaction between CAFs and cancer cells and for investigating potential therapeutic strategies to overcome CAF‐mediated resistance in cancer treatment. In future, mechanisms underlying the interaction between PANC‐1 and NIH/3 T3 cells, in terms of drug and radiation sensitivity, should be fully elucidated.

## AUTHOR CONTRIBUTIONS

**Yeon Ju Kim:** Conceptualization; investigation; methodology; validation; visualization; writing – original draft. **Hyeon Song Lee:** Conceptualization; investigation; methodology; validation; visualization; writing – original draft. **Dohyun Kim:** Conceptualization; investigation; methodology; validation; visualization; writing – original draft. **Hwa Kyung Byun:** Conceptualization; funding acquisition; supervision; writing – review and editing. **Woong Sub Koom:** Conceptualization; funding acquisition; supervision; writing – review and editing. **Won‐Gun Koh:** Conceptualization; funding acquisition; supervision; writing – review and editing.

## CONFLICT OF INTEREST STATEMENT

The authors have no conflicts of interest to declare.

### PEER REVIEW

The peer review history for this article is available at https://www.webofscience.com/api/gateway/wos/peer-review/10.1002/btm2.10708.

## Supporting information


**Movie S1.** Movie showing the separation of collagen/polyethylene glycol (PEG) hydrogel from alginate hydrogel.

**Data S1.** Supporting Information.

**Table S1.** Up‐regulated differentially expressed proteins (DEPs) of NIH/3 T3 co‐cultured with pancreatic cancer cells (PANC‐1) compared with mono‐cultured NIH/3 T3.

## Data Availability

Data are available from the corresponding author upon reasonable request.
