# Supplementary material for: Bilayer 3D co‐culture platform inducing the differentiation of normal fibroblasts into cancer‐associated fibroblast like cells: New in vitro source to obtain cancer‐associated fibroblasts
Source: Bioeng Transl Med. 2024 Aug 5;10(1):e10708. doi: 10.1002/btm2.10708 (PMC11711222; doi:10.1002/btm2.10708)
Supplement: Supplementary file 2 — Data S1. Supporting Information. [file BTM2-10-e10708-s001.docx]

**[Supplementary Materials]**

Bilayer 3D co-culture platform inducing the differentiation of normal fibroblasts into cancer-associated fibroblast like cells: New in vitro source to obtain cancer-associated fibroblasts

Yeon Ju Kim^2, +^, Hyeon Song Lee^1, +^, Dohyun Kim^1, +^, Hwa Kyung Byun^3^, Woong Sub Koom^2^, and Won-Gun Koh^1^

^1^ Department of Chemical and Biomolecular Engineering, Yonsei University, Seoul, South Korea

^2^Department of Radiation Oncology, Yonsei Cancer Center, Yonsei University College of Medicine, Seoul, South Korea

^3^Department of Radiation Oncology, Yongin Severance Hospital, Yonsei University College of Medicine, Yongin, South Korea

+ These authors contributed equally

* Corresponding author:

Prof. Hwa Kyung Byun, Department of Radiation Oncology, Yongin Severance Hospital, Yonsei University College of Medicine, Yongin, South Korea, E-mail: HKBYUN05@yuhs.ac

Prof. Woong Sub Koom, Department of Radiation Oncology, Yonsei Cancer Center, Yonsei University College of Medicine, Seoul, South Korea, E-mail: MDGOLD@yuhs.ac

Prof. Won-Gun Koh, Department of Chemical and Biomolecular Engineering, Yonsei University, Seoul, 03722, South Korea, E-mail: wongun@yonsei.ac.kr

**Supplementary materials contain 6 figures, 1 table (excel file), and 1 video**

**(mp4 file) supplied separately.**

**Figure S1**. Storage modulus and loss modulus of the alginate hydrogel (A) and PEG/collagen hydrogel (B).

**Figure S2**. Fluorescence image of physically isolated NIH/3T3 and PANC-1 co-cultured for 1 and 7 days. NIH-3T3 in collagen/PEG hydrogel was labeled with QD525 (green) and PANC-1 in alginate hydrogel was labeled with QD655(red). Scale bars correspond to 200 μm.

.

**Figure S3**. Effect of collagenase on the viability of NIH/3T3. A) Results of CCK-8 assay for 3 days and comparison of relative viability with control experiment. ***p < 0.001. (B) Live/dead fluorescence images of retrieved NIH/3T3. Images were obtained 3 days after re-seeding retrieved NIH/3T3. Scale bars correspond to 50 μm.

**Figure S4.** Comparison of proliferation between mono-cultured PANC-1 and co-cultured PANC-1. The proliferation of PANC-1 was analyzed by CCK-8 assay.

**Figure S5.** Verification of mRNA expression levels of CAF-related markers in NIH/3T3 cells co-cultured with 3D PANC-1 conditioned media. α-SMA and FAP mRNA levels were determined by real-time polymerase chain reaction (RT-PCR).

**Figure S6**. LC/MS proteomic analysis. (A) Venn diagram representing the number of differential expression proteins (DEPs). (B) Heat map image of DEPs (FC ≤ 2 or ≥0.5, p-value ≤ 0.05)

**Table S1**. Up-regulated DEPs of NIH/3T3 co-cultured with PANC-1 compared with mono-cultured NIH/3T3.

**Movie S1.** Movie showing the separation of collagen/PEG hydrogel from alginate hydrogel.

**
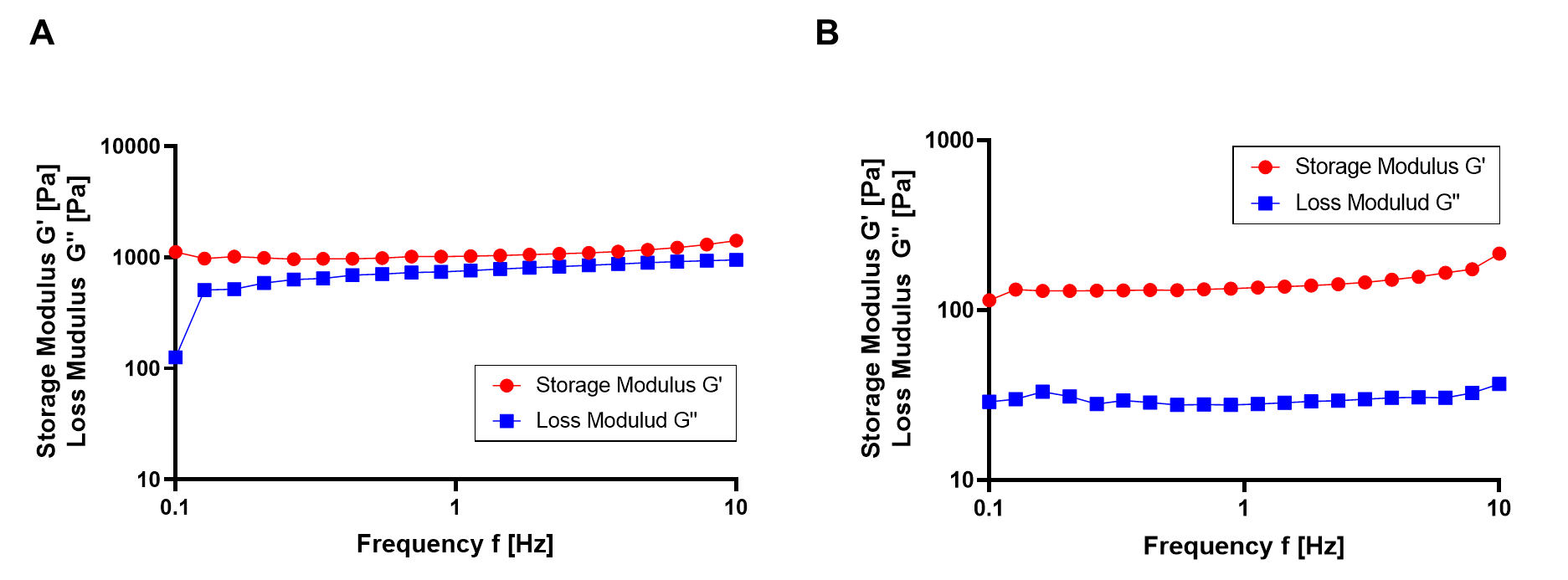
**

**Figure S1**. Storage modulus and loss modulus of the alginate hydrogel (A) and PEG/collagen hydrogel (B).


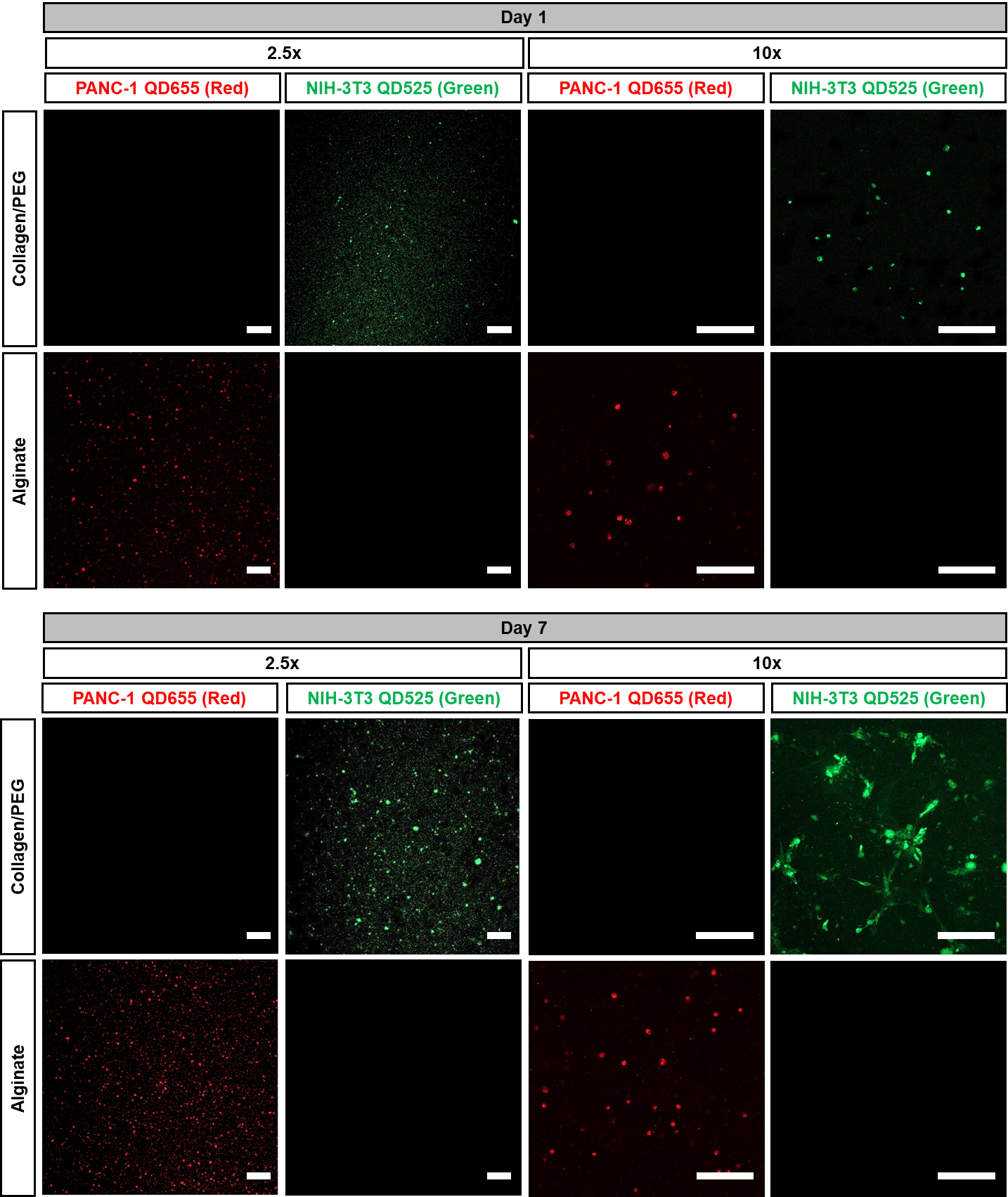


**Figure S2.** Fluorescence image of physically isolated NIH/3T3 and PANC-1 co-cultured for 1 and 7 days. NIH-3T3 in collagen/PEG hydrogel was labeled with QD525 (green) and PANC-1 in alginate hydrogel was labeled with QD655(red). Scale bars correspond to 200 μm.

Here, Panc-1 and NIH/3T3 remained inside the alginate and PEG/collagen hydrogel, respectively where they were originally encapsulated, and were not found within the other hydrogel even after 7 days.

**
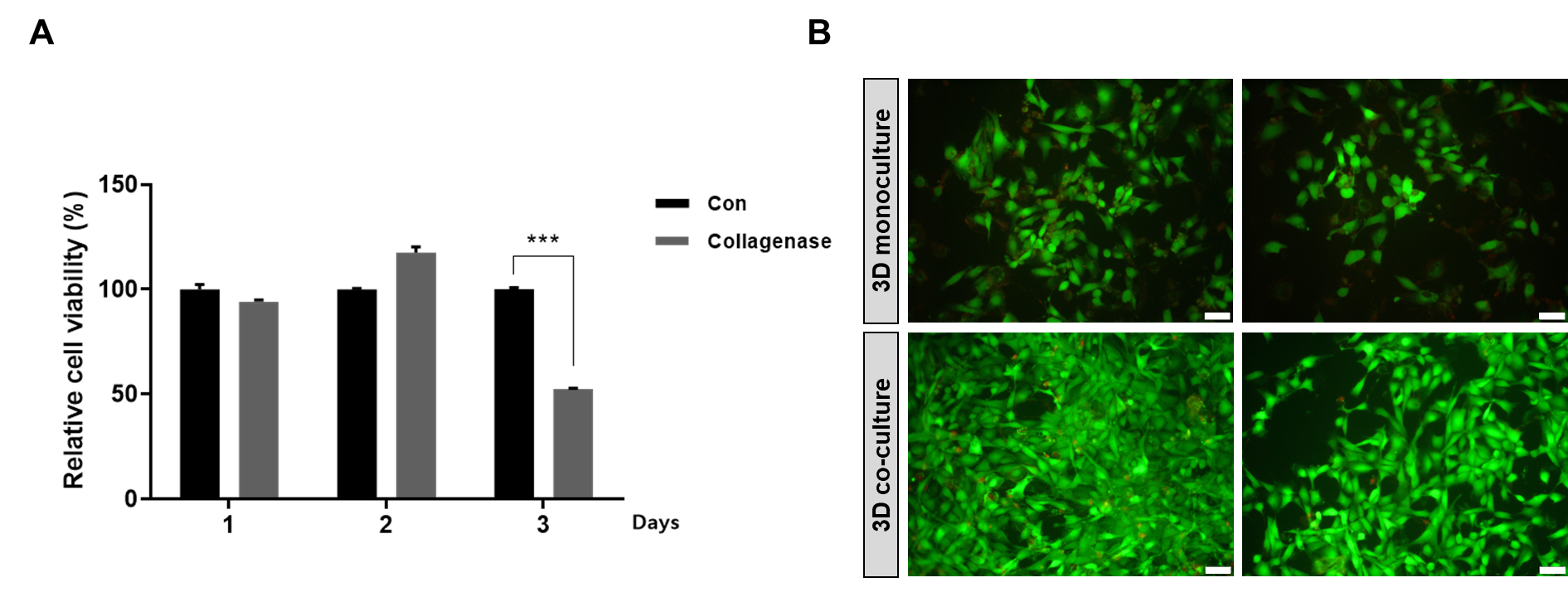
**

**Figure S3**. Effect of collagenase on the viability of NIH/3T3. A) Results of CCK-8 assay for 3 days and comparison of relative viability with control experiment. ***p < 0.001. (B) Live/dead fluorescence images of retrieved NIH/3T3. Images were obtained 3 days after re-seeding retrieved NIH/3T3. Scale bars correspond to 50 μm.


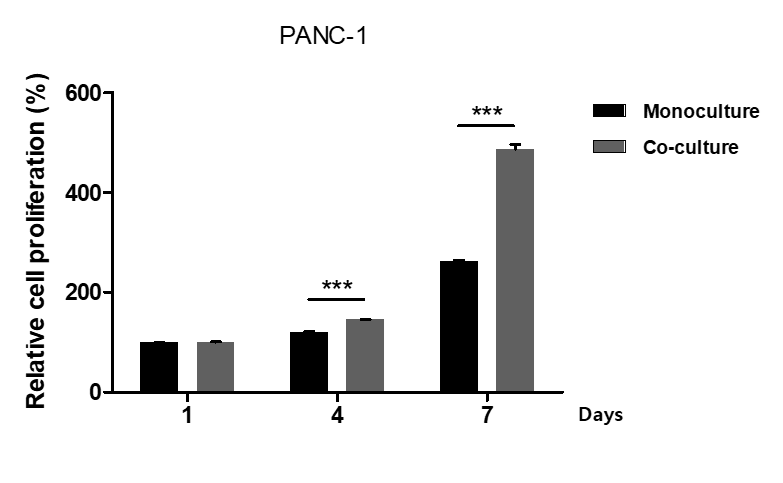


**Figure S4.** Comparison of proliferation between mono-cultured PANC-1 and co-cultured PANC-1. The proliferation of PANC-1 was analyzed by CCK-8 assay.


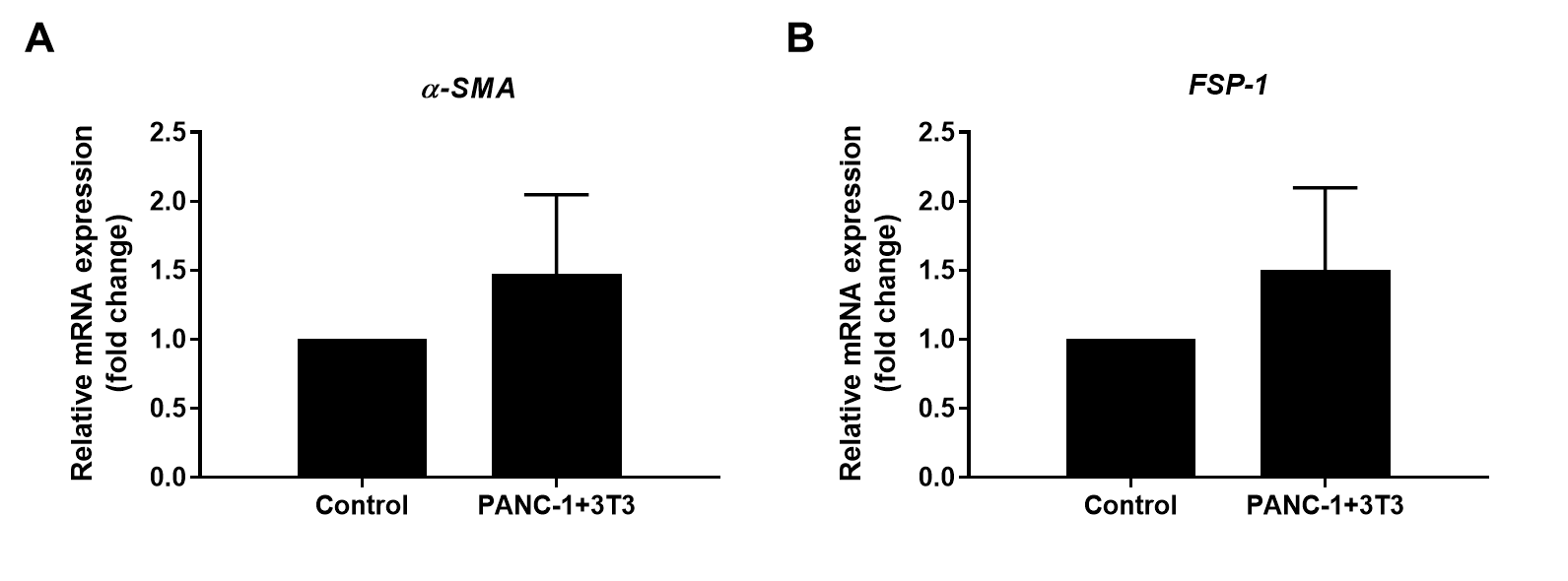


**Figure S5.** Verification of mRNA expression levels of CAF-related markers in NIH/3T3 cells co-cultured with 3D PANC-1 conditioned media (A: α-SMA and B: FSP-1) . α-SMA and FSP-1 mRNA levels were determined by real-time polymerase chain reaction (RT-PCR).


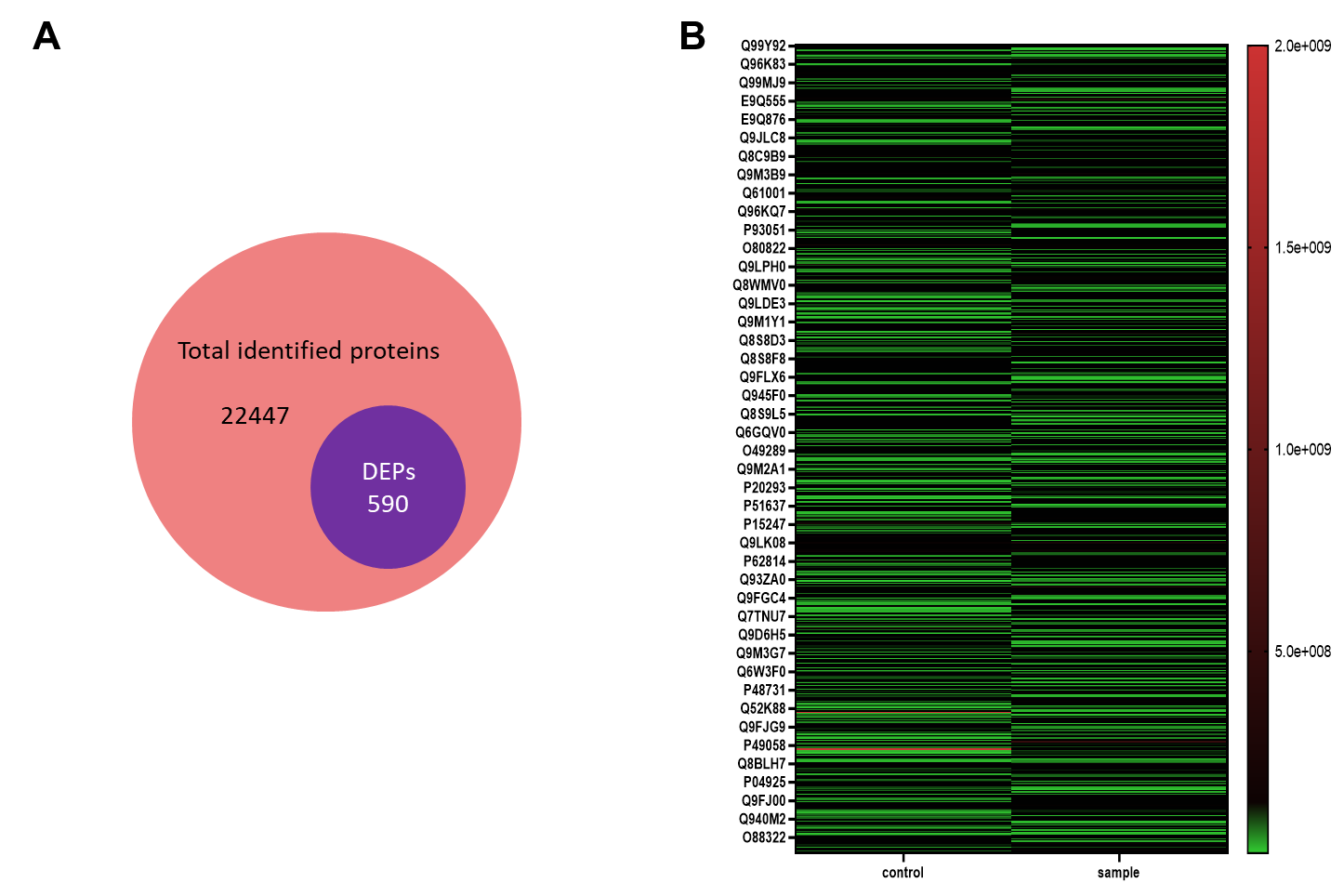


**Figure S6**. LC/MS proteomic analysis. (A) Venn diagram representing the number of differential expression proteins (DEPs). (B) Heat map image of DEPs (FC ≤ 2 or ≥0.5, p-value ≤ 0.05)
